# Cytoglobin expression in the hepatic stellate cell line HSC-T6 is regulated by extracellular matrix proteins dependent on FAK-signalling

**DOI:** 10.1186/s13069-015-0032-y

**Published:** 2015-08-21

**Authors:** Louise Catherine Stone, Lorna Susan Thorne, Christopher John Weston, Mark Graham, Nikolas John Hodges

**Affiliations:** School of Biosciences and School of Medicine, The University of Birmingham, Edgbaston, Birmingham, B15 2TT UK; School of Biosciences and MG Toxicology Consulting Ltd, Birmingham, UK

**Keywords:** Cytoglobin, Fibrosis, Hepatic stellate cell, Liver, Focal adhesion kinase

## Abstract

**Background:**

Fibrosis is a physiological response to cellular injury in the liver and is mediated by the activation of hepatic stellate cells resulting in the replacement of hepatocytes with extracellular matrix comprised principally of collagen 1 to form a hepatic scar. Although the novel hexaco-ordinated globin cytoglobin was identified in activated hepatic stellate cells more than 10 years ago, its role in stellate cell biology and liver fibrosis remains enigmatic.

**Results:**

In the current study, we investigated the role of different extracellular matrix proteins in stellate cell proliferation, activation (alpha smooth muscle actin expression and retinoic acid uptake) and cytoglobin expression. Our results demonstrate that cytoglobin expression is correlated with a more quiescent phenotype of stellate cells in culture and that cytoglobin is regulated by the extracellular matrix through integrin signalling dependent on activation of focal adhesion kinase.

**Conclusions:**

Although further studies are required, we provide evidence that cytoglobin is a negative regulator of stellate cell activation and therefore may represent a novel target for anti-fibrotic treatments in the future.

**Electronic supplementary material:**

The online version of this article (doi:10.1186/s13069-015-0032-y) contains supplementary material, which is available to authorized users.

## Background

Fibrogenesis is a physiological response to tissue insult or injury [1, 2] and is characterised by increased synthesis and deposition of extra cellular matrix (ECM) proteins, which can account for up to 22 % of the surface area of a fibrotic liver [3]. During liver fibrosis, the composition of the ECM changes from one dominated by collagen IV and laminin [4], to one of fibrillar collagen, predominantly collagen I and III, as well as fibronectin [5]. The primary source of ECM in the liver is hepatic stellate cells (HSCs) [6] and following liver injury; HSCs undergo phenotypic changes from quiescent vitamin A storing cells, involved in maintenance of the normal basement matrix, into proliferative myofibroblast-like cells, expressing myogenic markers such as alpha smooth muscle actin (αSMA). Activated HSCs synthesise and secrete collagens type I and III into the ECM [7] which takes place in concert with changes to the surrounding ECM. This could be considered to be part of a feedback loop as changes in ECM composition results in changes in HSC cell surface receptors, particularly integrins indicating that the ECM should not be regarded simply as an inert physical scaffold but a dynamic structure that is able to influence the phenotype of HSCs [2, 8, 9]. HSCs express numerous cell surface receptors including both discoidin domain receptors (DDRs)—which are receptors for collagen I and many of the integrin subunits, which are a major family of cell surface receptors for extracellular molecules and play a critical role in many biological functions [7, 10]. Interaction with ECM is a major driver of HSC morphology [11], and signal transduction from integrin activation occurs at least partly through aggregation and subsequent activation of focal adhesion kinases (FAKs) by autophosphorylation of tyrosine 397 (Y397). There are numerous downstream signalling targets of FAK with many of the effects ultimately mediated by changes in gene expression [11–13].

Cytoglobin (Cygb) was identified as a phenotypic marker of activation of rat HSCs following treatment in vivo with the pro-fibrotic agent carbon tetrachloride [14] and basal levels of expression have also been reported in HSCs [15]. Further work has shown that in the liver, Cygb expression is probably restricted to HSCs [15–17] suggesting a specific function for Cygb in the process of fibrosis. Although it is known that other globins including neuroglobin and myoglobin play an important role in oxygen homeostasis and metabolism [18–22], the specific biochemical function(s) of Cygb remains elusive. There is evidence that like other globins Cygb may have a role in the detoxification of reactive oxygen and nitrogen species (ROS, RNS) [23], and consistent with this hypothesis, purified Cygb has peroxidase activity, but it is not clear if this is physiologically relevant [15, 24]. Cygb may also be involved in intracellular oxygen storage and transport, with a possible role in facilitating transportation of oxygen to mitochondria to support the electron transport chain and oxidative phosphorylation [25, 26]. A fundamental problem with many of these ideas is the low tissue levels of expression of Cygb and restriction of expression to cells of a fibroblast lineage suggesting a more specific cellular function related to fibrosis in organs such as the liver.

Cygb up regulation during fibrogenesis occurs in parallel with changes in collagen expression in both the liver and the kidney [17, 27, 28], and Cygb knockout mice show reduced levels of liver fibrosis and ECM deposition following insult with carbon tetrachloride. Together, these data suggest a role for Cygb in the regulation of collagen synthesis by stellate cells during fibrosis in both of these organs. Cygb is also up regulated in the liver by hypoxia [16, 17, 29, 30], and it is known that cycles of hypoxia followed by re-oxygenation are important in the fibrotic processes of several organs including the lung, kidney, and liver [31–34]. It has been suggested that Cygb may be able to sense and direct cellular responses to these local changes in oxygen partial pressure to afford protection form hypoxic reperfusion injury. In support of this hypothesis, studies have shown a cyto-protective function for Cygb in vitro [35–38]. Furthermore, in the kidney, overexpression of Cygb in transgenic rats inhibited fibrosis and preserved renal function [36]. However, in this study, deposition of collagen and activation of myofibroblasts were reduced suggesting that in vivo, there may not be a direct relationship between Cygb expression and collagen deposition. In support of this, Nishi et al. [37] made similar observations under conditions of ischemia/reperfusion in the kidney.

Whilst a definitive function of Cygb remains to be discovered, these studies suggest that expression of the protein is intimately linked to the fibrotic process within the liver. In the current study, we have investigated the effect of different ECM proteins relevant to liver fibrosis on activation state and Cygb expression in hepatic stellate cells and shown for the first time that Cygb expression is regulated by components of the ECM and that this is mediated by changes in integrin signalling.

## Results

### Effect of ECM on cell morphology and proliferation

**Fig. 1:**
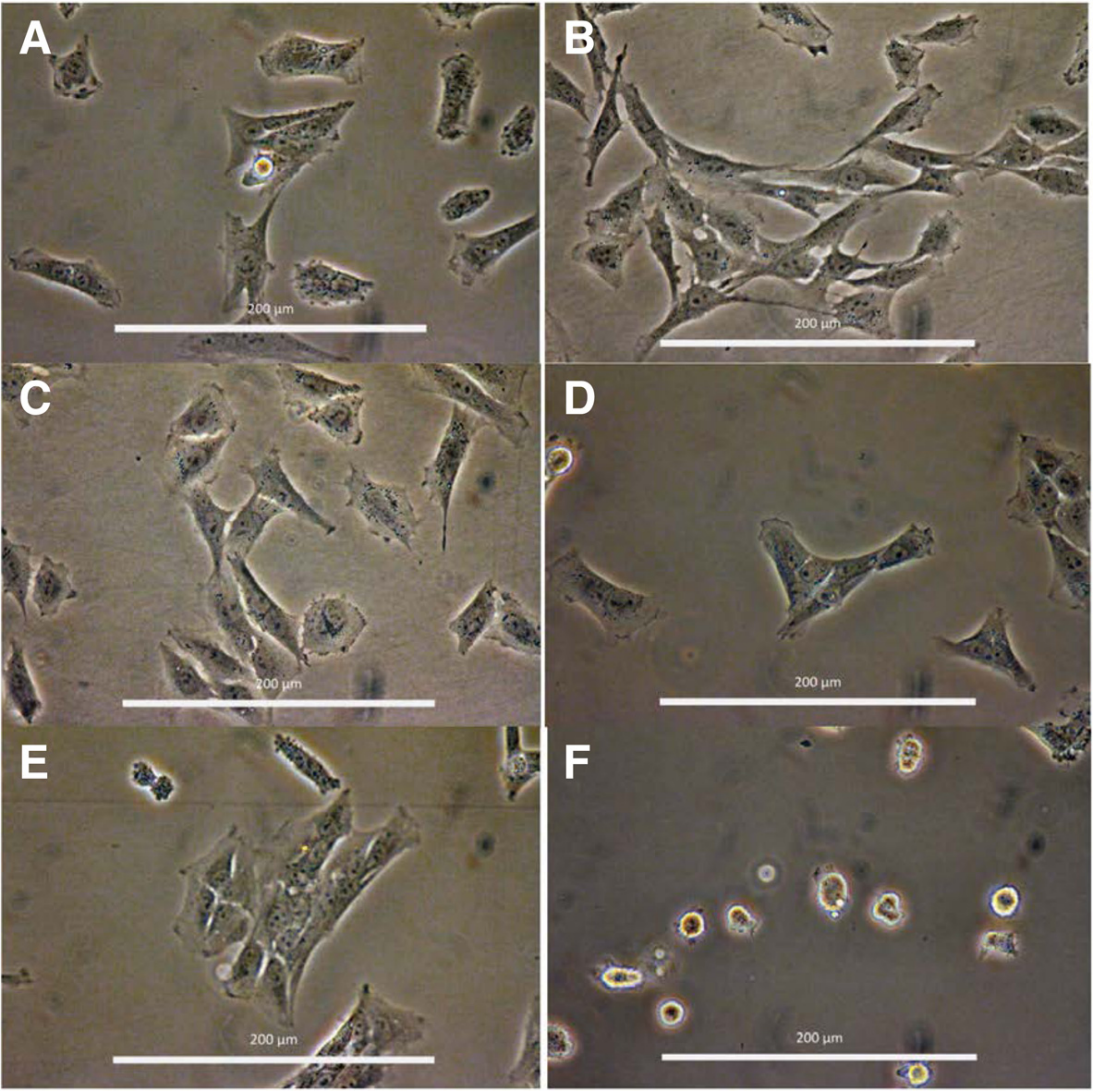
HSC-T6 cell morphology when cultured on different ECM proteins. Cells were imaged 24 h post seeding at 400× magnification. Enhanced using the one step Photo Fix function on Jasc Paint Shop Pro V 9.00. **a** non-coated, **b** gelatin, **c** collagen I, **d** collagen IV, **e** fibronectin and **f** laminin

**Fig. 2:**
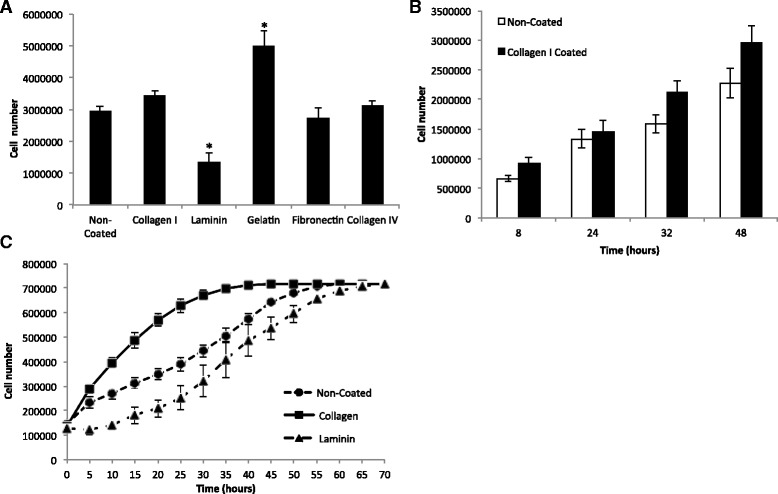
**a** Effect of ECM different proteins on the growth of HSC-T6 cells; *Denotes significant difference from non-coated control at *P* < 0.05 according to one-way ANOVA with Tukey’s post hoc *t* test. Cells were seeded at 100,000 cells/ml and counted on a haemocytometer 48 h post seeding. Cell density is expressed as cells per T_25_ flask. **b** Effect of ECM on cell number per T_25_ across a passage (48 h). Cells from the same population were seeded at 100,000 cells/ml, 5 ml per flask and grown on non-coated and collagen I coated flasks. Cells were counted on a haemocytometer at four different time points. The results represent the mean of three experiments ± SD. Not significant (one way ANOVA *P* = 0.34). **c** Cell number per well over 70 h. Cells from the same population were seeded at 100,000 cells/ml, 1 ml per well in a 24 well plate and grown on non-coated plastic, laminin or collagen I. Cell number was quantified by the Cell-IQ analysis software. Cell number on laminin was significantly different (*P* < 0.01 one-way ANOVA) from cell number on non-coated plastic or on collagen I between 5–45 h of culture, with the exception of hour 25. Number of cells on collagen I were significantly different from cell number on non-coated plastic between 15 and 40 h. The results represent the mean of three experiments ±SD

Visual inspection of HSC-T6 cells 24 h after seeding clearly showed that culture on gelatin (denatured collagen), collagen, or fibronectin had no effect on the visual appearance of HSC-T6 cells when compared to cells cultured on uncoated plastic, with all cells exhibiting an elongated, fibroblast-like shape (Fig. 1a–e). In contrast, culture of cells on laminin had a marked effect, with adherent cells exhibiting a more rounded morphology suggestive of reversion to a more quiescent state (Fig. 1f). Cells grown on laminin also reached a statistically significant (*P* < 0.05) lower cell density after 48 h of culture compared to uncoated controls (Fig. 2). In contrast, there was no statistically significant difference in cell number for cultures grown on fibronectin compared to uncoated controls. After 48 h culture on gelatin, there was a statistically significant increase in cell number compared with the uncoated control (*P* < 0.05), and although not statistically significant, there was also a trend of increased cell number for cells grown on both collagen I and IV protein (Fig. 2a). To further investigate the effect of collagen I and laminin on proliferation of HSC-T6 cells, cell numbers were quantified at different time points over the period of a single passage. As quantified using a haemocytometer (Fig. 2b), cell number for cells cultured on collagen I was consistently higher than controls at all time points investigated (8, 24, 32, and 48 h). Furthermore, detailed analysis of cellular proliferation by Cell-IQ clearly showed that the initial rates of cell proliferation were in the order collagen I > uncoated control > laminin and that the time taken for the initial two cell doublings were approximately 10, 25, and 35 h, respectively (Fig. 2c). Despite the difference in initial rates of cellular growth, cells reached a final level of confluence of approximately 700,000 cells irrespective of the ECM protein that they were cultured on. However, this took 45 h for cells on collagen I, 55 h for the uncoated control, and 65 h for cells grown on laminin (Fig. 2c).

**Fig. 3:**
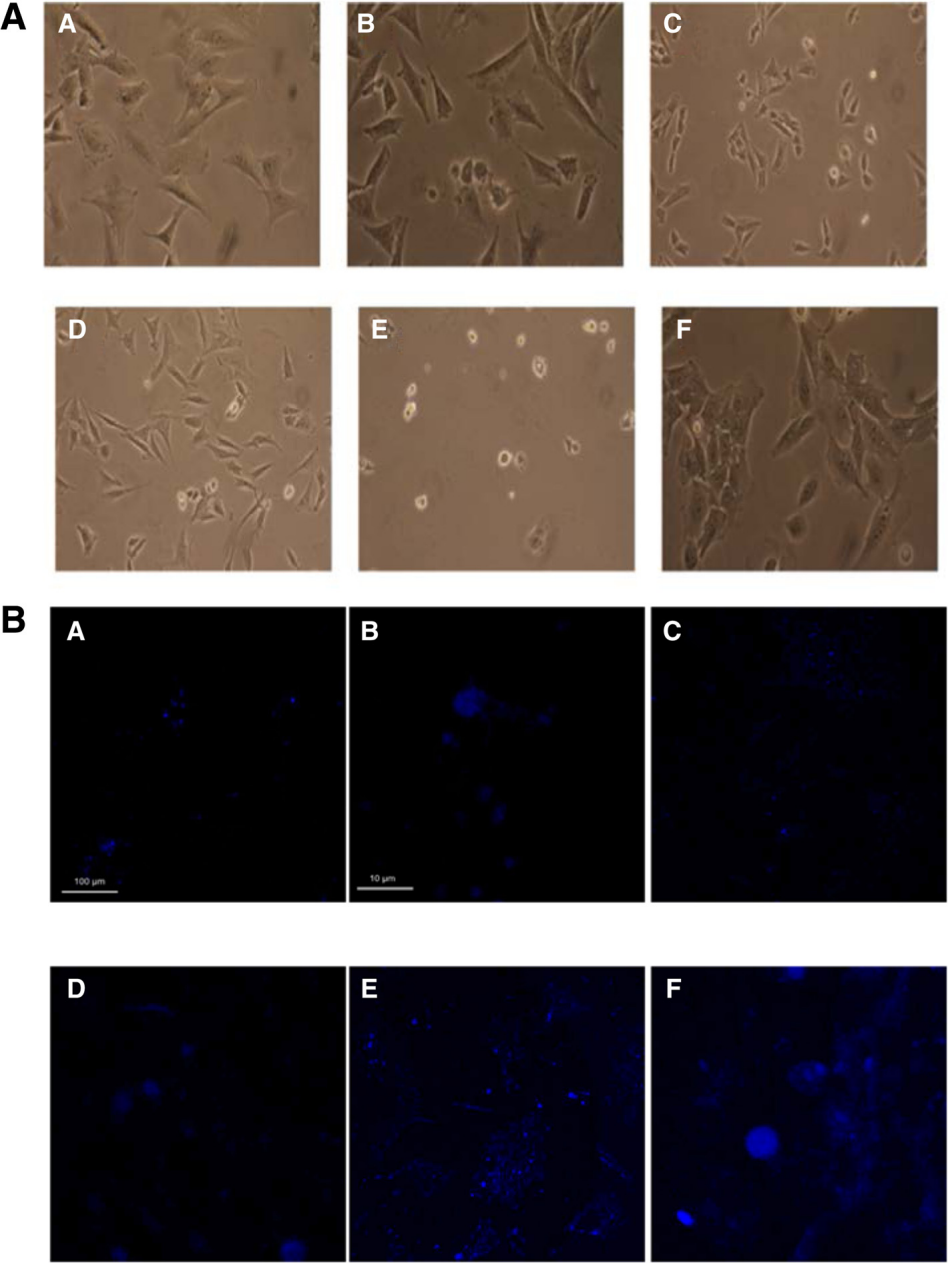
**a** Images of HSC-T6 cells cultured on mixed matrix surfaces of collagen I and laminin in differing concentrations 48 h post seeding at 100,000 cell/ml at 400× magnification. **a** 10:0 μg/cm^2^, **b** 10:5 μg/cm^2^, **c** 10:10 μg/cm^2^, **d** 5:10 μg/cm^2^, **e** 0:10 μg/cm^2^ collagen I: laminin and **f** non-coated control. **b** Confocal images of HSC-T6 cells seeded at 100,000 cells/ml on glass, collagen I and laminin coverslips 48 h post seeding, blue retinoid autofluorescence activated at 351 nm and detected at 515 nm. **a** glass, **b** glass zoom, **c** collagen I, **d** collagen I zoom, **e** laminin, **f** laminin zoom. Image analysis in ImageJ showed a statistically significant 5-fold increase in the fluorescence of cells cultured on laminin in the presence ATRA compared to untreated controls (*P* < 0.001) from 5.15 ± 2.7 to 27.8 ± 12 relative fluorescence units respectively

In vivo, HSCs are exposed to an environment containing a mixture of different ECM proteins, the composition of which is known to alter depending on the physiological status of the liver, being dominated by laminin in normal basement membranes and replaced by collagen I during fibrosis. Therefore, we investigated the effect of different ratios of laminin and collagen I on HSC-T6 morphology. Interestingly, the visual appearance of HSC-T6 cells was dominated by the presence of collagen I with the phenotype essentially the same as on collagen I alone even in the presence of a 2-fold excess of laminin (Fig. 3a). Next, we investigated the effect of ECM protein on uptake of all trans retinoic acid (ATRA), a marker of differentiation in hepatic stellate cells. Consistent with our findings above, only cells cultured on laminin were able to uptake ATRA (Fig. 3b). Cellular uptake of ATRA appeared to be largely restricted to cytoplasmic vesicles and image analysis using ImageJ indicated a statistically significant (*P* < 0.001) 5-fold increase in retinoid autofluorescence from 5.15 ± 2.7 to 27.8 ± 12 relative fluorescence units following treatment with ATRA compared to controls. Cultures of the human HSC cell line (LX-2) revealed a similar but less marked change in appearance when cells were cultured on laminin. Like HSC-T6 cells, no change in morphology was observed when cultured on collagen I (Additional file 1: Figure S1). Furthermore, there was no statistically significant change in end point cell counts after 48 h of culture (Additional file 2: Figure S2A). Analysis by Cell-IQ showed that the initial rates of cell proliferation were in the order collagen I = uncoated control > laminin and that the time taken for two cell doublings were approximately 10 h for uncoated controls and collagen I and 20 h for cells grown on laminin (Additional file 2: Figure S2B). Like HSC-T6 cells, the LX-2 line was also able to reach a final level of confluence of approximately 700,000 cells irrespective of the ECM protein that they were grown on. This took 25 h for cells on the uncoated surface and collagen I but 35 h for the cells grown on laminin (Additional file 2: Figure S2B). In addition, ATRA uptake in LX-2 cells was consistent with results observed in HSC-T6 cells, with only cells cultured on laminin showing detectable uptake of ATRA (Additional file 3: Figure S3).

### ECM regulates αSMA and Cygb expression

**Fig. 4:**
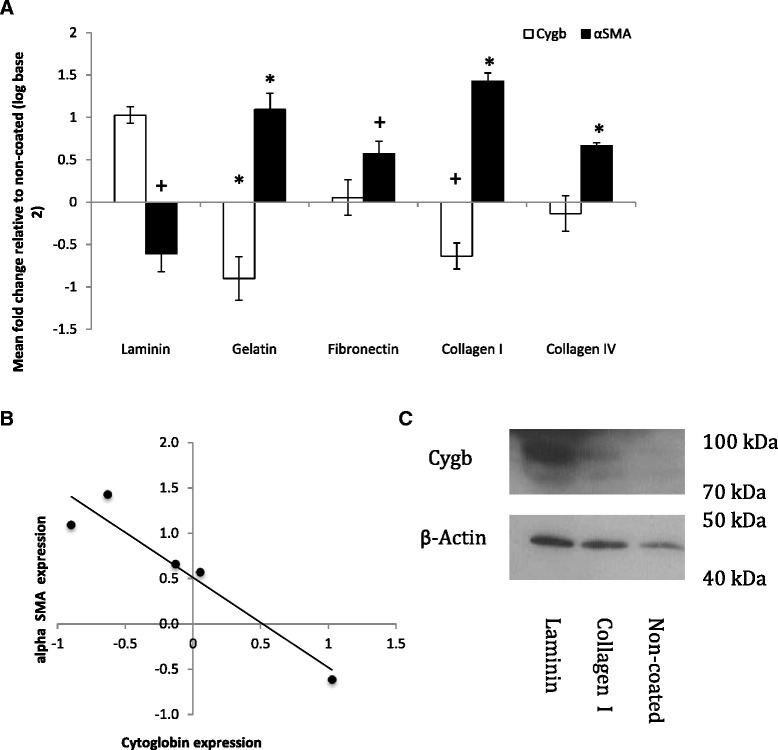
**a** Cygb and αSMA expression in HSC-T6 cells cultured on different ECM proteins analysed by RT-qPCR calibrated to expression on non-coated Flasks. *Denotes significant difference from non-coated control at *P* < 0.05 according to one-way ANOVA with Tukey’s post hoc test; + denotes significant difference from non-coated control at *P* < 0.05 according to *t* test assuming equal variances. The results represent the mean of three experiments ± SD. **b** Statistically significant (*R* ^2^ = 0.913, *P* < 0.05) negative correlation between αSMA and Cygb expression. **c** Western blot analysis of Cygb protein expression on non-coated plastic, collagen I and laminin

The activation status of HSC-T6 cells was determined by quantifying αSMA mRNA expression, a marker of stellate cell activation [7], and was found to be significantly altered by culture on different ECM proteins. αSMA expression was elevated in cells cultured on collagen I, collagen IV, fibronectin, and gelatin compared to culture on uncoated plastic (*P* < 0.05). In contrast, expression was decreased in cells cultured on laminin (*P* < 0.05, Fig. 4a). The expression of Cygb was also altered by culture on different ECM proteins and, interestingly, changes in Cygb expression were reciprocal to those observed for αSMA expression, being down regulated or unchanged on collagen I, collagen IV, and gelatin and increased in cells cultured on laminin compared with uncoated plasticware as a control (Fig. 4a), with a statistically significant negative correlation between Cygb and αSMA expression (*R*^2^ = 0.913, *P* < 0.05, Fig. 4b). An increase in Cygb expression in HSC-T6 cells cultured on laminin was confirmed at the protein level by Western blotting (Fig. 4c).

**Fig. 5:**
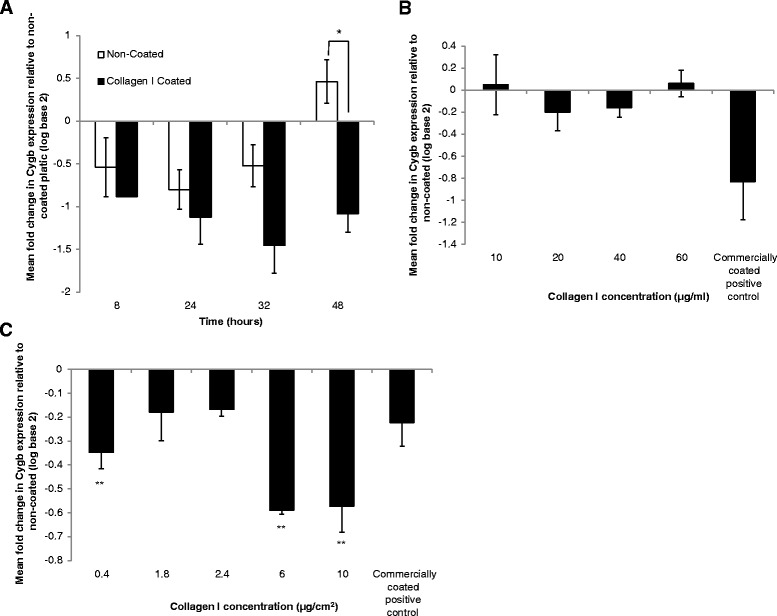
**a** Expression of Cygb across a passage (48 h) on non-coated and collagen I coated flasks relative to 0 h time point on non-coated plastic. *Denotes significant difference at *P* < 0.05 according to Mann-Whitney *U* test. The results represent the mean of three experiments ± SD. **b** Expression of Cygb in HSC-T6 in the presence of collagen I 48 h after seeding on non-coated plastic with collagen I diluted into the culture media relative to Cygb expression on non-coated plastic. The results represent the mean of three experiments ±SD. **c** Cygb expression in cells cultured on collagen I coated plates at different concentrations expressed relative to that of Cygb cultured on non-coated plastic. **Denotes significant difference (*P* < 0.01) from non-coated control according to one-way ANOVA with Tukey’s post hoc. The results represent the mean of three experiments ±SD

Next, we investigated the time dependency of Cygb expression in HSC-T6 cells across a single 48 h passage. Interestingly, when compared to Cygb expression in cultures immediately after attachment, expression of Cygb was reduced in cells cultured on both plastic and collagen I after 8, 24, and 32 h of culture, but this effect was more pronounced in cells cultured on collagen I. However, in confluent cells, after 48 h of culture, Cygb expression returned to levels slightly above initial values in cells cultured on uncoated plastic but remained significantly reduced in cells cultured on collagen I (Fig. 5a). To investigate whether changes in Cygb expression were dependent on attachment of cells to a collagen I, we tested if collagen in suspension could also reduce Cygb expression. As shown in Fig. 5b, culture of cells in media containing collagen I (0–60 μg/ml) in solution had no effect on Cygb expression. Rather, attachment to collagen was a requirement for reduced Cygb expression in HSC-T6 cells. This effect was statistically significant and most marked at concentrations of collagen I greater than 6 μg/cm^2^ (Fig. 5c). Comparable experiments in the human LX-2 cell line demonstrated that Cygb expression was also down regulated in this cell line when cultured on collagen I (Additional file 4: Figure S4). However, in contrast to HSC-T6 cells, there was no statistically significant effect of laminin on Cygb expression in LX-2 cells (Additional file 4: Figure S4).

**Fig. 6:**
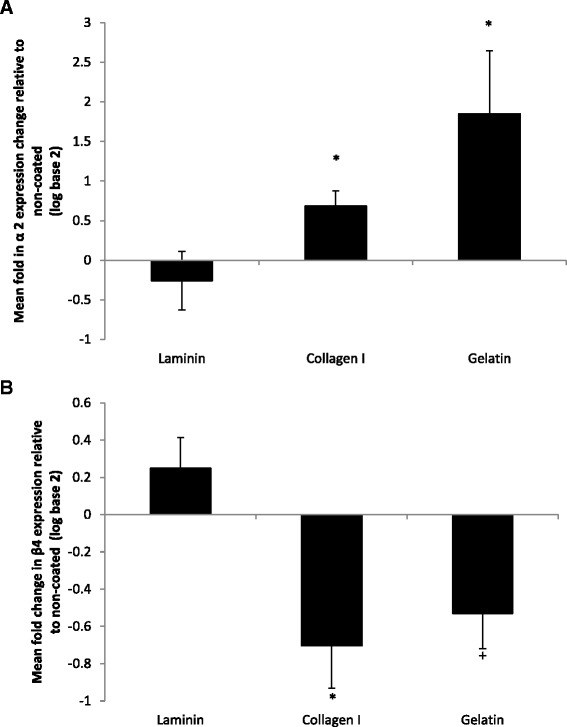
Expression of **a** α2 and **b** β4 integrin subunit ECM receptors following culture of HSC-T6 on laminin, collagen I and gelatin ECM proteins relative to the expression levels of those grown on non-coated T_25_ culture flasks. *Denotes significant difference from non-coated control at *P* < 0.05 according to one-way ANOVA with Tukey’s post hoc test; + denotes significant difference from non-coated control at *P* < 0.05 according to *t* test assuming equal variances. The results represent the mean of three experiments ±SD

### FAK-dependent regulation of Cygb and intracellular ROS

**Fig. 7:**
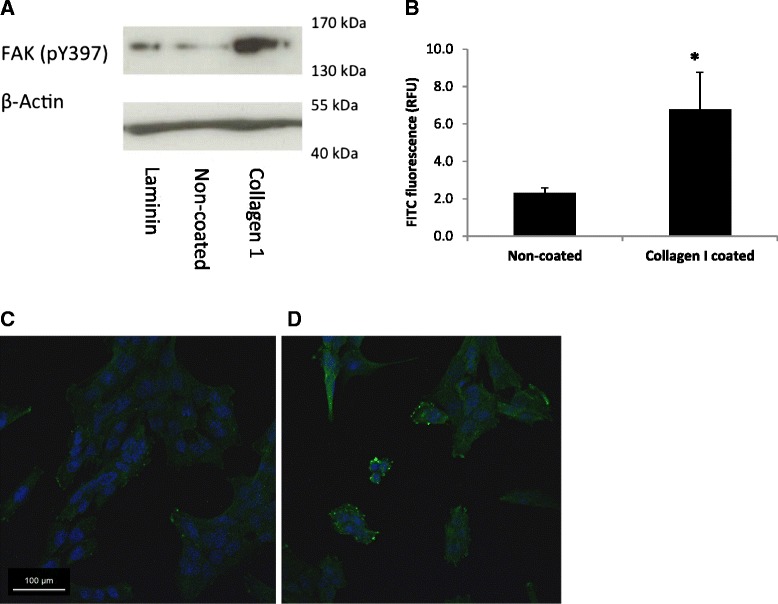
Analysis of FAK Y397 phosphorylation in HSC-T6 cells cultured different ECM surfaces for 48 h. **a** Western blot for protein expression of phosphorylated FAK Y397 in cells cultured on laminin, non-coated tissue culture plastic and collagen I with β-Actin loading control. **b** Mean FAK Y397 FITC fluorescence detected by flow cytometry in HSC-T6 cells cultured on non-coated plastic and collagen I; * denotes significant difference (*P* < 0.01). The results represent the mean of three experiments ±SD. **c**, **d** Confocal image of cells cultured on glass and collagen I and probed for expression of phosphorylated FAK Y397 (*green*). Nuclei are shown in *blue* (Hoechst 33342)

To investigate the signalling pathways likely to be responsible for ECM modulation of Cygb expression in HSC-T6 cells, we quantified the level of expression of integrin receptors (Additional file 5: Table S1) by qPCR. Out of a panel of 7 integrin receptors and 2 discoidin receptors (DDR1 and 2), only integrin receptor subunits α2 and β4 showed statistically significant (*P* < 0.05) ECM protein dependent changes in expression (Fig. 6). The other receptors quantified (α5, α6, α11, β1, β3, DDR1, DDR2) showed no significant changes in expression. Interestingly, integrin α2 expression was significantly up regulated in cells cultured on both collagen I and gelatin proteins and down regulated in cells grown on laminin (Fig. 6a). In contrast, the opposite pattern of expression was observed for the β4 subunit (Fig. 6b). To further support a possible role for integrin receptor signalling, we investigated the effect of ECM on focal adhesion kinase (FAK) activation. Increased phosphorylation (Y397) and activation were observed in cells cultured on collagen I compared to plastic as assessed by Western blotting (Fig. 7a), flow cytometry (Fig. 7b) and confocal microscopy (Fig. 7c, d). In contrast, little to no activation of FAK was observed when cells were cultured on laminin (Fig. 7a).

**Fig. 8:**
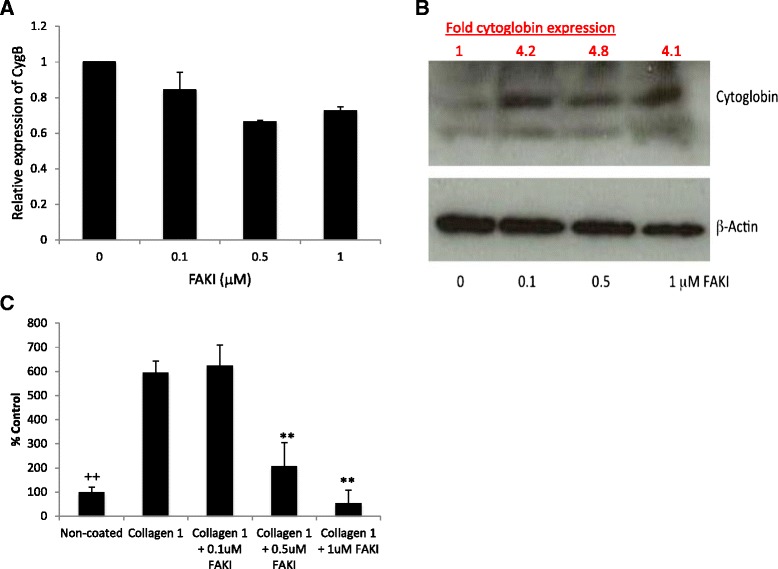
Effect of incubation of cells with the FAK-inhibitor FAKI 14 on levels of Cygb as assessed by **a** qPCR and **b** Western blotting. **c** Induction of ROS as assessed by fluorescein oxidation in cells cultured on a collagen I surface and its inhibition by treatment with FAKI 14. The results represent the mean of three experiments carried out in triplicate ±SD. ++ and ** are significantly different from uncoated and untreated collagen control *P* < 0.01, respectively (one-way ANOVA with Tukey’s post hoc *t* test)

In order to test directly the hypothesis that regulation of Cygb expression by collagen I is FAK dependent, cells were treated with a FAK-inhibitor (FAKI14) and levels of Cygb quantified by qPCR and Western blotting. After preliminary experiments to identify non-toxic concentrations of FAKI14 (Additional file 6: Figure S5), it was determined that incubation of cells with FAKI14 (1 μM) for the final 24 h of a 48-h culture effectively inhibited collagen-I-induced phosphorylation of FAK as assessed by both flow cytometry and confocal microscopy (Additional file 7: Figure S6). Next levels of Cygb expression following treatment of cells cultured on a collagen I surface and treated with FAKI 14 were quantified. As shown in Fig. 8, although there was a small decrease in levels of Cygb mRNA, this was not statistically significant (Fig. 8a). However, in support of our hypothesis, a concentration-dependent increase in levels of Cygb protein was observed in cells treated with FAKI 14 (Fig. 8b). Interestingly, we also observed that culture of cells on collagen I induced levels of ROS, which is generated in cells following activation of FAK-signalling, and this was also inhibited in cells cultured in the presence of FAKI (Fig. 8c).

**Fig. 9:**
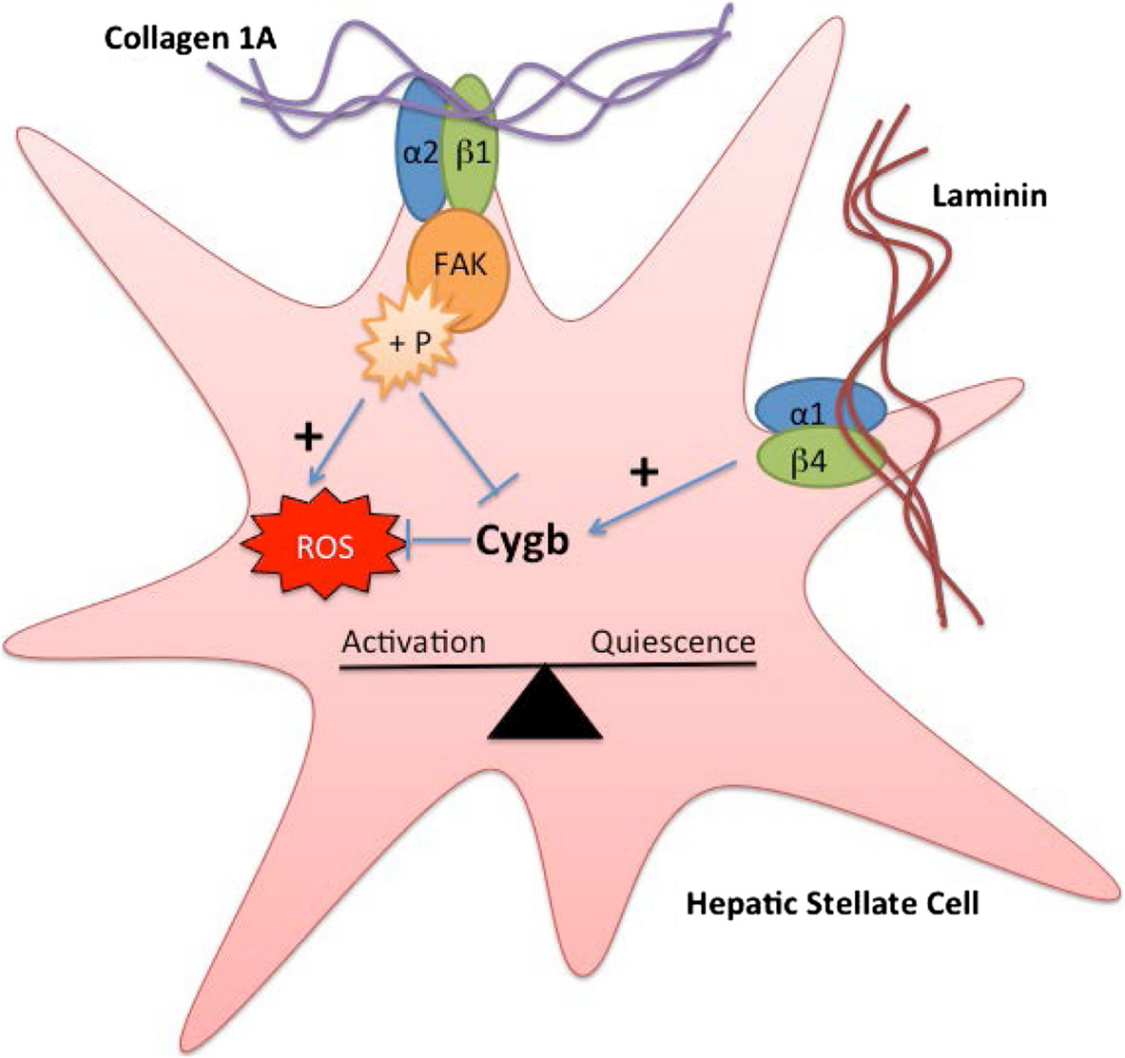
Proposed relationship between ECM proteins, cytoglobin expression and stellate cell activation. Extracellular collagen 1A stimulates FAK phosphorylation via integrin signalling (most likely α2β1) resulting in elevated levels of intracellular ROS and decreased cytoglobin expression leading to stellate cell activation. In contrast, extracellular laminin causes increases in cytoglobin expression possibly via integrin signalling resulting in a more quiescent phenotype

## Discussion

The role of ECM proteins in stellate cell biology have been studied previously, for example Davis et al. [39] reported that cell surface substrates modulate stellate cell behaviour, collagen production and response to retinoids. Friedman et al. [40] have also examined the effect of basement membrane matrix on stellate cell phenotype. In agreement with these findings, we observed that when compared with cells grown on uncoated plastic, HSC-T6 cells cultured on collagen and gelatin (denatured collagen) proliferate more quickly and had a more activated phenotype as assessed by increased expression of αSMA. In contrast, cells grown on laminin although maintaining the ability to proliferate albeit more slowly in culture had what appeared to be a more quiescent phenotype when assessed by the same marker. The ability of HSC-T6 cells to uptake ATRA, another marker of quiescence in stellate cells [41] only when grown on laminin provides further evidence of a more quiescent phenotype. Priya and Sudhakaran [42] have also investigated the effect of ECM proteins on primary rat stellate cells and observed that cells cultured on collagen I lose the ability to take up retinol, produce greater amounts of glycosaminoglycans and are more resistant to curcumin-induced apoptosis when compared with those cultured on either collagen IV or laminin. HSC-T6 resemble semi-activated primary rat HSC as they express both markers of activation such as αSMA but also share characteristics in common with freshly isolated quiescent rat HSCs [43]. Interestingly, there is an in vivo precedent for partially activated stellate cells. A study by Kisseleva et al. [44] has shown that following fibrotic injury, a subpopulation of HSCs is not removed by apoptosis as previously thought but remain in the liver in what is considered a partially active state although their precise biological function remains to be determined. The ability to change HSC-T6 cells to a more quiescent state by culturing on a laminin protein is a novel observation and could be used to improve existing in vitro models of stellate cell activation and biology. Importantly, the effect of laminin on proliferation of LX-2 cells and uptake of ATRA was broadly consistent with results in HSC-T6 cells suggesting a genuine biological response rather than an artefact of a specific cell culture model. Although beyond the scope of the current study, ultimately it would be desirable to confirm our data in primary hepatic stellate cells.

To our knowledge this is the first study to report that Cygb expression is differentially regulated in HSCs by different ECM proteins, being down regulated by both gelatin and collagen I, which is a major constituent of the hepatic scar, and up regulated on laminin, which is a major component of the basement membrane matrix in a normal liver. Down regulation of Cygb expression in HSC-T6 cells by extracellular collagen was also observed in LX-2 cells. Conversely, in HSC-T6 cells, Cygb expression was up regulated by extracellular laminin. Together, these data support the hypothesis that Cygb expression in HSCs is regulated by the composition of the extracellular environment. This is interesting because HSCs are the major architects of changes to ECM proteins during the process of fibrotic liver injury in vivo, and our data suggests that signalling via the ECM provides a potential feedback mechanism to regulate stellate cell function and that Cygb is a downstream target of altered cell signalling in this process.

Our observation that gelatin also negatively regulates Cygb expression is intriguing. Although gelatin is not widely considered as a physiological molecule, studies have suggested that HSCs may come into contact with a form of denatured collagen similar to gelatin during the resolution of fibrosis [9, 45]. Gelatin does not bind to and activate either of the main discoidin domain receptors (DDR1 and DDR2) as the triple helix of collagen is required for DDR activation [46], whereas in contrast, integrins recognise short amino acid motifs in ligands, such as Arg-Gly-Asp, as binding sites and may potentially be activated by gelatin [47]; this may also explain our observation that proliferation of HSC-T6 cells is induced by both collagen 1 and gelatin, although the reason for the quantitative difference in this effect remains to be determined. In the current study, consistent with previous reports [46, 7], both DDR1 and DDR2 were expressed in HSC-T6 cells. However, culture on different cell surface proteins had no effect on levels of their expression; therefore, it seems unlikely that either DDR1 or 2 are responsible for the observed changes in Cygb expression. In contrast, our observation of up regulation of integrin α2 expression and down regulation of integrin β4 on collagen and the reciprocal change produced by laminin suggest that an integrin signalling pathway is involved in regulating changes in Cygb expression in HSC-T6 cells. It is known that integrin dimers containing the α2 subunit (e.g., α2β1) are receptors for collagen 1 [48, 49], and it seems likely therefore that changes in α2β1 integrin signalling mediate the changes in cellular phenotype of HSC-T6 cells cultured on collagen I and rates of proliferation when grown on either collagen 1 or gelatin. Similarly, integrin dimers containing the β4 subunit are known laminin receptors, and it is likely that these may mediate changes in response to culture on laminin.

We also observed that phosphorylation of FAK occurred in cells cultured on collagen I, but not laminin, further supporting a role for integrin signalling in regulation of Cygb expression although the role of FAK-signalling in regulating cell proliferation and αSMA expression in HSC-T6 cells is unknown and requires further investigation. One of the cellular consequences of activation of integrin signalling is the generation of reactive oxygen species (ROS), which is important for downstream signal transduction [50]. It is believed that this effect is important in a number of pathological conditions including fibrosis [51–53] and is mediated by the GTPase Rac1 that orchestrates a number of changes resulting in the production of ROS including alteration of mitochondrial function and activation of NADPH-oxidases (NOX). It has been demonstrated that persistent activation of Rac1 in hepatic stellate cells can promote both fibrosis and liver injury in vivo [54]. Consistent with these findings, in the current study, we report increases in levels of intracellular ROS following culture of cells on collagen 1 and activation of FAK, and levels of ROS could be inhibited by incubation of cells with a FAK-inhibitor. Similarly, collagen-I-dependent down regulation of Cygb was also inhibited in cells treated with FAK-inhibitor. There is evidence that Cygb can function as an intracellular anti-oxidant [37, 38, 55–57] including in hepatic stellate cells [57] and that Cygb deficiency has been linked to elevated liver cancer via a mechanism involving hepatosteatosis dependent on oxidative stress [58]. We therefore hypothesise that down regulation of Cygb is either directly or indirectly responsible for the increase in levels of intracellular ROS observed. The mechanism of FAK-dependent regulation of Cygb expression remains to be determined, but our data suggests that this may occur at both the transcriptional and posttranscriptional level because treatment of cells with FAKI had no statistical significant effect of levels of Cygb mRNA despite elevated protein levels. It may be possible for example that following FAK inhibition degradation of Cygb protein may be inhibited although this requires further study.

The data reported in the current study also supports the conclusion that Cygb should be considered a marker of HSC quiescence because of its negative correlation with αSMA expression, a classic marker of stellate cell activation [7]. Although in this study we did not investigate levels of αSMA protein, previous studies have shown a good correlation between levels of αSMA mRNA and protein indicating that measurement of αSMA at the mRNA level alone is sufficient to demonstrate stellate cell activation [59, 60]. Furthermore, elevated Cygb expression by laminin also correlated with increased cellular uptake of ATRA, reduced cellular proliferation and a more quiescent phenotype. These findings are initially surprising as there is an extensive literature that supports the hypothesis that Cygb expression is a marker that positively correlates with stellate cell activation in vivo [15, 61]. However, in agreement with the finding presented here, more recent studies using Cygb transgenic animals to study kidney fibrosis observed that Cygb inhibits fibrosis and preserves renal function [36]. The same study also reported that deposition of collagen and activation of myofibroblasts was reduced by Cygb expression, suggesting that in vivo there may be a feedback loop between deposition of collagen in the ECM and stellate cell activation. Based on our in vitro data in HSC-T6 cells, we hypothesis that this is mediated by α2β1 integrin signalling, FAK activation and the subsequent generation of ROS that is further facilitated by reduction of expression of Cygb.

In further support of our findings and their in vivo relevance, Man et al. [17] have shown an increase in Cygb expression in the livers of mice subjected to an acute CCl_4_ toxic insult at 24 h. The expression of Cygb then decreased to below that found in normal liver at the 48-h time point, which coincided with an increase in collagen IαI. Further evidence that Cygb may be associated with a more quiescent stromal phenotype comes from the study of Cui et al [62], which reported that primary rat HSCs treated with arundic acid showed a marked increase in Cygb RNA and protein expression and a decrease in αSMA expression. Le et al. [63] have also presented evidence that Cygb may be anti-fibrotic, where Cygb knockout mice showed increased hepatosteatosis when fed a choline-deficient amino acid defined diet, probably through increased ROS and cytokine production. These reports agree with our in vitro observations of a reciprocal relationship between Cygb expression and stellate cell activation. Our data also provides the basis of a mechanistic explanation of these in vivo data and suggests that this effect may be mediated by the response of stellate cells to their extracellular environment—specifically collagen I via integrin cell signalling as summarised in Fig. 9.

## Conclusions

In conclusion, the current study demonstrates a role for integrin-dependent FAK-signalling in the regulation of Cygb expression in stellate cells grown on a collagen I surface and regulation of levels of intracellular ROS. This may be important in the fibrotic process and the development of liver disease, as the ECM proteins which regulate Cygb expression, laminin and collagen I, are important components of the basement matrix in a normal liver and of the hepatic scar, respectively. Although further mechanistic studies are required there is emerging evidence that Cygb may represent a novel target for anti-fibrotic treatments in the future.

## Methods

### Cell culture

HSC-T6, an immortalised rat hepatic stellate cell line first described by Friedman et al [64] were a gift from Francis Chang (Institute of Cancer Research, Royal Cancer Hospital). LX-2 cells, an immortalised human hepatic stellate cell line first described by Xu et al [65] were a gift from Christopher Weston (The University of Birmingham). Both cell lines were maintained at 37 °C, 5 % CO_2_/air in high (4.5 g/L) glucose Dulbecco’s Modified Eagle’s Medium (Sigma-Aldrich) supplemented with either 2 % (LX-2) or 10 % (HSC-T6) (*v*/*v*) foetal bovine serum (FBS), penicillin (100 units/ml), streptomycin (100 μg/ml) (PAA), 2 mM L-glutamine (PAA) and 1 % (*v*/*v*) MEM non-essential amino acids (Sigma-Aldrich). For routine culture, cells were passaged twice weekly using a standard trypsin-EDTA protocol.

### Extracellular matrix experiments

Both cell lines were plated at a density of 500,000 per flask into T_25_ flasks coated with either collagen I, collagen IV, laminin, fibronectin (BD BioCoat) or gelatin (coated overnight with a 0.1 % *v*/*v* solution). Uncoated plasticware was used as a negative control (BD Biosciences). Cells were cultured for 48 h and then passaged into a new flask with the same surface coating at a density of 500,000 cells/T_25_ flask, for the duration of three passages. After 48 h of passage three, cells were trypsinised and cell number quantified using a haemocytometer. In other experiments, cell number was quantified over a time course. Briefly, cells were seeded at 500,000 cells per T_25_ flask and cell number was determined using a haemocytometer after 8, 24, 32 and 48 h or quantified in real time using a Cell-IQ (CM Technologies) over a 72-h period. In additional experiments collagen I solution from rat tail (Sigma) was either diluted into culture media at 10, 20, 40 or 60 μg/ml or used to coat plates at 0.4, 0.8, 1.8, 2.4, 6 and 10 μg/cm^2^. Cells were then cultured for 48 h and total RNA extracted for qPCR analysis as described below. To investigate possible cellular interactions with both collagen I and laminin, six well plates were coated with different amounts of collagen I and laminin (ultrapure BD) as follows—10:0, 10:5, 10:10, 5:10 and 0:10 μg/cm^2^ collagen I: laminin. Cells were then seeded at a density of 200,000 cells per 6-well plate and cultured for 48 h. At the end of the experiment, the cells were photographed and total RNA and protein were isolated for analysis by qPCR or Western blotting analysis. Protein extracts, 15 μl, (approx. 20 μg) determined by the method of Bradford [66], were prepared in Laemmli loading buffer (0.42 M SDS, 0.87 mM bromophenol blue, 47 % *v*/*v* glycerol, 60 mM Tris pH 6.8 and 1.6 M β-mercaptoethanol) and resolved on a 12.5 % SDS-PAGE electrophoresis gel, transferred to PVDF and probed for Cygb (Santa-Cruz, clone FL-190, 1:200 dilution). Equal loading was confirmed by blotting with β-actin (Sigma, 1:10,000 dilution), secondary antibody (goat anti-rabbit or goat anti-mouse HRP, DAKO) was used at 1:500 or 1:1000 and the signal was visualised using ECL femto reagent (Geneflow) and an X-ograph (AGFA Curix60). For light microscopy, cells were imaged using a Nikon Eclipse TS100 light microscope ×40 objective and a Canon EOS 7D digital camera. All images were processed in an identical manner using the one step Photo Fix function on Jasc Paint Shop Pro V 9.00.

### Retinoic acid uptake

Round 22 mm glass coverslips were coated with 10 μg/cm^2^ of either collagen I or laminin, and cells were seeded at a density of 200,000 per well in a 6-well plate containing the pre-coated coverslip; an uncoated coverslip was used as a negative control. Once the cells had adhered, all trans retinoic acid (ATRA, Sigma, 1 μM, prepared in DMSO) was added to the culture media and cells were cultured for 48 h. Cells were fixed in 4 % paraformaldehyde (pH 7.4) and coverslips were mounted onto microscope slides with hydromount (Fisher Scientific). Cells were imaged using a Leica TCS SP2 confocal microscope with a HCX PL APO 63x/1.40-0.60 oil immersion objective. The excitation wavelength was 351 nm, and emitted light was collected between 515 and 550 nm. All images were processed in an identical manner using Adobe Photoshop CC 2014 and analysed using ImageJ (NIH, Bethesda; version 1.48).

### Treatment of cells with FAK-Inhibitor and analysis of phosphorylated FAK

After identification of non-toxic concentrations of FAKI14 by the 3-(4,5-dimethylthiazol-2-yl)-2, 5-diphenyltetrazolium (MTT) assay [67], levels of phosphorylated FAK were determined by Western blotting, confocal microscopy and flow cytometry. In all experiments, cells were seeded at 200,000 cells per 6-well plates either uncoated or collagen coated (BD Biocoat). For Western blot, whole cell protein was extracted directly into Laemmli loading buffer. Protein extracts (30 μl) were resolved on a 12.5 % SDS-PAGE electrophoresis gel, transferred to PVDF and probed for tyrosine 397 phosphorylated FAK (BD clone, 14/FAK (Y397) 1:500 dilution). Equal loading was confirmed by blotting with β-actin (Sigma, 1:10,000 dilution), secondary antibody (goat anti-mouse HRP, DAKO) was used at 1:1000 in both instances. Signal was visualised by ECL Pico reagent (Thermo) using an X-ograph (AGFA Curix60).

For confocal microscopy, cells were cultured as before, fixed in 4 % paraformaldehyde and permeabilised in 90 % methanol then probed for FAK pY397 (1:50) followed by FITC-conjugated secondary (goat anti-mouse, DAKO 1:20). Nuclei were counterstained with Hoechst 33342 (0.6 μM). Cells were imaged using a Leica TCS SP2 confocal microscope and a HCX PL APO 63x/1.40-0.60 oil immersion objective. The excitation wavelength was 405 nm for Hoechst 33342 and 488 nm for FITC. All images were processed in an identical manner using Adobe Photoshop CC 2014.

For analysis by flow cytometry, cells were detached from the plate by trypsinisation for 10 min, pelleted by centrifugation and re-suspended in 2 ml of blocking buffer (3 % FBS in PBS). Cells were washed twice in blocking buffer and then incubated in 100 μl blocking buffer for 10 min with agitation. pFAK Y397 antibody (BD Bioscience) was added to a final concentration of 1 μg/ml, and samples were incubated at room temperature for 1 h. Samples were then rinsed by centrifugation as before, then 100 μl of FITC (fluorescein isothiocyanate) conjugated anti-mouse 2°Ab (Dako, UK) diluted 1:20 in incubation buffer was added and samples were incubated in the dark for 30 min at room temperature with agitation. Cells were then rinsed as before and re-suspended in 500 μl PBS. FITC fluorescence of 10,000 cells was analysed using BD FACScalibur™ (BD Bioscience) and Weasel software (Walter and Elisa Hall Institute of Medical Research, Australia) was used for data analysis.

### Intracellular ROS assay

Cells were seeded at 200,000 cells per well on either uncoated or collagen-coated 6-well plates (BD Biocoat) and grown for 48 h in the presence or absence of FAKI (0–1 μM) for the final 24 h of incubation. Cells were then incubated with 2′,7′-dichlorodihydrofluorescein diacetate (H_2_DCF-DA, 10 μM) for 2 h, then trypsinised for 10 min at 37 °C to obtain a single cell suspension after which the cells were pelleted by centrifugation for 3 min at 900 g. Cells were re-suspended in PBS (1 ml), and FITC fluorescence of 10,000 live cells was analysed using a BD FACScalibur™ (BD Bioscience), and Weasel software (Walter and Elisa Hall Institute of Medical Research, Australia) was used for data analysis.

### RNA Isolation and quantitative real-time PCR

For all experiments, total RNA was isolated from cells using a Qiagen RNeasy Miniprep Kit including a DNase I step. RNA was quantified on a Nanodrop ND1000 and 0.5 μg used for cDNA synthesis, using an Agilent Affinity Script Multitemp cDNA synthesis kit and oligo (dT) primers in a final volume of 20 μl. Real-Time PCR was conducted using 1 μl cDNA, 1 μl Primer-Probe (Applied Biosystems or Primer Design) and 10 μl qPCR Brilliant III Ultra-Fast QPCR MasterMix (Agilent), made up to 20 μl with RNase Free Water. The RT-PCR reaction conditions were 1 cycle at 95 °C for 3 min then 50 cycles of 95 °C for 20 s then 60 °C for 20 s, during which the data was gathered; details of all primers used are shown in Additional file 5: Table S1.

### Statistical analysis

Quantitative real-time PCR was analysed using the Pfaffl method [68]. Primer efficiencies were estimated using either a standard curve or linReg PCR software. Data was assessed for normality and then analysed using one-way ANOVA, Student’s *t* test, Kruskall-Wallis or Mann-Whitney *U* tests. Data are shown as mean ± SD.

## Additional files

Additional file 1: Figure S1. LX-2 cell morphology on different ECM proteins. Cells were seeded at 300,000 cells/ml imaged 48 hrs post seeding at 100x magnification. A) Non-Coated. B) Collagen I. C) Laminin. (DOCX 729 kb) Additional file 2: Figure S2. Proliferation of LX-2 cells on different ECM proteins. A) Cells were counted using a haemocytometer and counts were taken at 48 hrs post seeding. Cell density is expressed as cells per T_25_ flask. The results represent the mean of three experiments ± SD. B) Cells from the same population were seeded at 300,000 cells/ml, 1 ml per well and grown on non-coated plastic, laminin or collagen I. Cell number was estimated by the Cell-IQ analysis software. The results represent the mean of three experiments ± SD. (DOCX 53.1 kb) Additional file 3: Figure S3. Confocal Images of LX-2 cells treated with ATRA cultured on glass, collagen I and laminin 48 hrs post seeding at 300,000 cells/ml, blue—retinoid autofluorescence activated at 351 nm and detected at 515 nm. A) glass, B) collagen I, C) collagen I zoom, D) laminin, E) laminin zoom. (DOCX 260 kb) Additional file 4: Figure S4. Expression of Cygb in LX-2 cells cultured on collagen I and laminin proteins analysed by RT-qPCR calibrated to expression on non-coated flasks. * denotes significant difference from non-coated plastic at p = 0.05 according to Kruskall-Wallis. The results represent the mean of three experiments ± SD. (DOCX 33.9 kb) Additional file 5: Table S1. Details Of hydrolysis probe based qPCR assays used in this study. (DOCX 40.6 kb) Additional file 6: Figure S5. MTT viabilty assay for HSC-T6 cells cultured on non-coated tissue culture plastic and collagen I and treated with FAK-Inhibitor 14. Cell viability given as a % of the non-treated control for each surface, * denotes significance at the p < 0.01 level according to One-Way ANOVA with Tukey’s Post-Hoc test. The results represent the mean of three experiments ± SD. (DOCX 575 kb) Additional file 7: Figure S6. A) Median flouresence of FAK pY397 analysed by flow cytomtery in cells treated with different concentrations FAKI 14. Cells were seeded at 100,000 cells/ml cultured for 24 hrs prior to 24 hrs of treatment with FAK I then probed for FAK pY397. The results represent the mean of three experiments ± SD. B) Confocal Microscopy of cells probed for FAK pY397 cultured on non-coated (B-1-4) or collagen I coated (B-5-8) coverslips. Cells were either untreated (B-1 and B-5) or treated with 0.1 μM (B-2 and B-6), 0.5 μM (B-3 and B-7) or 1.0 μM (B-4 and B-8) FAKI 14. (DOCX 88.9 kb)

## Abbreviations

ATRA: all trans retinoic acid
Cygb: cytoglobin
DDRs: discoidin domain receptors
DMSO: dimethylsulfoxide
ECM: extra cellular matrix
FAKs: focal adhesion kinases
FITC: fluorescein isothiocyanate
H_2_DCF-DA: 2′,7′-dichlorodihydrofluorescein diacetate
HSCs: hepatic stellate cells
MTT: 3-(4,5-dimethylthiazol-2-yl)-2,5-diphenyltetrazolium
NOX: NADPH-oxidases
RNS: reactive nitrogen species
ROS: reactive oxygen species
αSMA: alpha smooth muscle actin

## References

[1] Reeves HL, Friedman SL (2002). Activation of hepatic stellate cells—a key issue in liver fibrosis. Front Biosci.

[2] Bedossa P, Paradis V (2003). Liver extracellular matrix in health and disease. J Pathol.

[3] Lin XZ, Horng MH, Sun YN, Shiesh SC, Chow NH, Guo XZ (1998). Computer morphometry for quantitative measurement of liver fibrosis: Comparison with Knodell’s score, colorimetry and conventional description reports. J Gastroenterol Hepatol.

[4] Martinez-Hernandez A, Amenta PS (1995). The extracellular matrix in hepatic regeneration. FASEB J.

[5] Friedman SL (2008). Hepatic fibrosis-overview. Toxicology.

[6] Gabele E, Brenner DA, Rippe RA (2003). Liver fibrosis: signals leading to the amplification of the fibrogenic hepatic stellate cell. Front Biosci.

[7] Friedman SL (2008). Hepatic stellate cells: protean, multifunctional, and enigmatic cells of the liver. Physiol Rev.

[8] Benyon RC, Iredale JP (2000). Is liver fibrosis reversible?. Gut.

[9] Friedman SL (2008). Mechanisms of hepatic fibrogenesis. Gastroenterology.

[10] Carloni V, Romanelli RG, Pinzani M, Laffi G, Gentilini P (1996). Expression and function of integrin receptors for collagen and laminin in cultured human hepatic stellate cells. Gastroenterology.

[11] Streuli CH (2009). Integrins and cell-fate determination. J Cell Sci.

[12] Clark EA, Brugge JS (1995). Integrins and signal-transduction pathways—the road taken. Science.

[13] Qin J, Vinogradova O, Plow EF (2004). Integrin bidirectional signaling: a molecular view. PLoS Biol.

[14] Kristensen DB, Kawada N, Imamura K, Miyamoto Y, Tateno C, Seki S (2000). Proteome analysis of rat hepatic stellate cells. Hepatology.

[15] Kawada N, Kristensen DB, Asahina K, Nakatani K, Minamiyama Y, Seki S (2001). Characterization of a stellate cell activation-associated protein (STAP) with peroxidase activity found in rat hepatic stellate cells (vol 276, pg 25318, 2001). J Biol Chem.

[16] Schmidt M, Gerlach F, Avivi A, Laufs T, Wystub S, Simpson JC (2004). Cytoglobin is a respiratory protein in connective tissue and neurons, which is up-regulated by hypoxia. J Biol Chem.

[17] Man KN, Philipsen S, Tan-Un KC (2008). Localization and expression pattern of cytoglobin in carbon tetrachloride-induced liver fibrosis. Toxicol Lett.

[18] Dickerson RE, Geis I (1983). Hemoglobin: structure, function, evolution, and pathology.

[19] Brunori M (1999). Hemoglobin is an honorary enzyme. Trends Biochem Sci.

[20] Minning DM, Gow AJ, Bonaventura J, Braun R, Dewhirst M, Goldberg DE (1999). Ascaris haemoglobin is a nitric oxide-activated ‘deoxygenase’. Nature.

[21] Merx MW, Flögel U, Stumpe T, Gödecke A, Decking UKM, Schrader J (2001). Myoglobin facilitates oxygen diffusion. FASEB J.

[22] Bunn FH, Forget BG (1986). Hemoglobin—molecular, genetic, and clinical aspects. Hemoglobin Mol Gen Clin Asp.

[23] Burmester T, Gerlach F, Hankeln T, Roach RC, Wagner PD, Hackett PH (2007). Regulation and role of neuroglobin and cytoglobin under hypoxia. Hypoxia and the Circulation. Advances in Experimental Medicine and Biology.

[24] Reeder BJ, Svistunenko DA, Wilson MT (2011). Lipid binding to cytoglobin leads to a change in haem co-ordination: a role for cytoglobin in lipid signalling of oxidative stress. Biochem J.

[25] Burmester T, Ebner B, Weich B, Hankeln T (2002). Cytoglobin: a novel globin type ubiquitously expressed in vertebrate tissues. Mol Biol Evol.

[26] Sawai H, Kawada N, Yoshizato K, Nakajima H, Aono S, Shiro Y (2003). Characterization of the heme environmental structure of cytoglobin, a fourth globin in humans. Biochemistry.

[27] Nakatani K, Okuyama H, Shimahara Y, Saeki S, Kim DH, Nakajima Y (2004). Cytoglobin/STAP, its unique localization in splanchnic fibroblast-like cells and function in organ fibrogenesis. Lab Invest.

[28] Xu R, Harrison PM, Chen M, Li L, Tsui TY, Fung PC (2006). Cytoglobin overexpression protects against damage-induced fibrosis. Mol Ther.

[29] Senoo H (2004). Structure and function of hepatic stellate cells. Med Electron Microsc.

[30] Fordel E, Thijs L, Moens L, Dewilde S (2007). Neuroglobin and cytoglobin expression in mice—evidence for a correlation with reactive oxygen species scavenging. Febs J.

[31] Nangaku M (2004). Hypoxia and tubulointerstitial injury: a final common pathway to end-stage renal failure. Nephron Exp Nephrol.

[32] Rosmorduc O, Housset C (2010). Hypoxia: a Link between fibrogenesis, angiogenesis, and carcinogenesis in liver disease. Semin Liver Dis.

[33] Jain M, Sznajder JI (2005). Effects of hypoxia on the alveolar epithelium. Proc Am Thorac Soc.

[34] Higgins DF, Kimura K, Iwano M, Haase VH (2008). Hypoxia-inducible factor signaling in the development of tissue fibrosis. Cell Cycle.

[35] Hodges NJ, Innocent N, Dhanda S, Graham M (2008). Cellular protection from oxidative DNA damage by over-expression of the novel globin cytoglobin in vitro. Mutagenesis.

[36] Mimura I, Nangaku M, Nishi H, Inagi R, Tanaka T, Fujita T (2010). Cytoglobin, a novel globin, plays an antifibrotic role in the kidney. Am J Physiol-Renal Physiol.

[37] Nishi H, Inagi R, Kawada N, Yoshizato K, Mimura I, Fujita T (2011). Cytoglobin, a novel member of the globin family, protects kidney fibroblasts against oxidative stress under ischemic conditions. Am J Pathol.

[38] McRonald FE, Risk JM, Hodges NJ (2012). Protection from intracellular oxidative stress by cytoglobin in normal and cancerous oesophageal cells. PLoS One.

[39] Ahern M, Hall P, Halliday J, Liddle C, Olynyk J, Ramm G (1996). Hepatic stellate cell nomenclature. Hepatology.

[40] Friedman SL, Roll FJ, Boyles J, Arenson DM, Bissell DM (1989). Maintenance of differentiated phenotype of cultured rat hepatic lipocytes by basement-membrane matrix. J Biol Chem.

[41] She HY, Xiong SG, Hazra S, Tsukamoto H (2005). Adipogenic transcriptional regulation of hepatic stellate cells. J Biol Chem.

[42] Priya S, Sudhakaran PR (2008). Cell survival, activation and apoptosis of hepatic stellate cells: modulation by extracellular matrix proteins. Hepatol Res.

[43] Vogel S, Piantedosi R, Frank J, Lalazar A, Rockey DC, Friedman SL (2000). An immortalized rat liver stellate cell line (HSC-T6): a new cell model for the study of retinoid metabolism in vitro. J Lipid Res.

[44] Kisseleva T, Cong M, Paik Y, Scholten D, Jiang CY, Benner C (2012). Myofibroblasts revert to an inactive phenotype during regression of liver fibrosis. Proc Natl Acad Sci U S A.

[45] Ramachandran P, Iredale JP. Liver fibrosis: a bidirectional model of fibrogenesis and resolution. QJM. 2012. doi:hcs069 [pii]10.1093/qjmed/hcs069. 10.1093/qjmed/hcs069 PMC3424469 22647759

[46] Vogel WF, Abdulhussein R, Ford CE (2006). Sensing extracellular matrix: an update on discoidin domain receptor function. Cell Signal.

[47] Humphries JD, Byron A, Humphries MJ (2006). Integrin ligands at a glance. J Cell Sci.

[48] Heino J (2000). The collagen receptor integrins have distinct ligand recognition and signaling functions. Matrix Biol.

[49] Leitinger B (2011). Transmembrane collagen receptors. Annu Rev Cell Dev Biol.

[50] Eble JA, de Rezende FF (2014). Redox-relevant aspects of the extracellular matrix and its cellular contacts via integrins. Antioxid Redox Signal.

[51] Shi-wen X, Thompson K, Khan K, Liu S, Murphy-Marshman H, Baron M (2012). Focal adhesion kinase and reactive oxygen species contribute to the persistent fibrotic phenotype of lesional scleroderma fibroblasts. Rheumatology (Oxford).

[52] Gonzalez-Ramos M, de Frutos S, Griera M, Luengo A, Olmos G, Rodriguez-Puyol D (2013). Integrin-linked kinase mediates the hydrogen peroxide-dependent transforming growth factor-beta1 up-regulation. Free Radic Biol Med.

[53] Park SA, Kim MJ, Park SY, Kim JS, Lee SJ, Woo HA (2015). EW-7197 inhibits hepatic, renal, and pulmonary fibrosis by blocking TGF-beta/Smad and ROS signaling. Cell Mol Life Sci.

[54] Choi SS, Sicklick JK, Ma Q, Yang L, Huang J, Qi Y (2006). Sustained activation of Rac1 in hepatic stellate cells promotes liver injury and fibrosis in mice. Hepatology.

[55] Fordel E, Thijs L, Martinet W, Lenjou M, Laufs T, Van Bockstaele D (2006). Neuroglobin and cytoglobin overexpression protects human SH-SY5Y neuroblastoma cells against oxidative stress-induced cell death. Neurosci Lett.

[56] Fang J, Ma I, Allalunis-Turner J (2011). Knockdown of cytoglobin expression sensitizes human glioma cells to radiation and oxidative stress. Radiat Res.

[57] Lu Y, Wang Q, Li Z, Diao Y, Xu R (2011). Role of cytoglobin in protecting hepatic stellate cells against oxidation induced damage. Sheng Wu Gong Cheng Xue Bao.

[58] le Thuy TT, Matsumoto Y, Thuy TT, Hai H, Suoh M, Urahara Y (2015). Cytoglobin deficiency promotes liver cancer development from hepatosteatosis through activation of the oxidative stress pathway. Am J Pathol.

[59] Fang L, Huang C, Meng X, Wu B, Ma T, Liu X (2014). TGF-β1-elevated TRPM7 channel regulates collagen expression in hepatic stellate cells via TGF-β1/Smad pathway. Toxicol Appl Pharmacol.

[60] Chen Y-XX, Weng Z-HH, Zhang S-LL (2012). Notch3 regulates the activation of hepatic stellate cells. World J Gastroenterol.

[61] Asahina K, Kawada N, Kristensen DB, Nakatani K, Seki S, Shiokawa M (2002). Characterization of human stellate cell activation-associated protein and its expression in human liver. Biochim Biophys Acta-Gene Struct Expression.

[62] Cui WH, Wang M, Maegawa H, Teranishi Y, Kawada N (2012). Inhibition of the activation of hepatic stellate cells by arundic acid via the induction of cytoglobin. Biochem Biophys Res Commun.

[63] Le TT, Suoh M, Matsumoto Y, Shimada M, Hirano Y, Motoyama H (2012). Cytoglobin deficiency promotes live fibrosis and liver cancer development in mice with steatohepatitis throughout activating oxidative stress pathway. Hepatology.

[64] Friedman SL, Lalazar A, Wong L, Blaner WS, Vogel S, Theiss G (1997). HSC-T6 cells, an immortalized rat hepatic stellate cell line. Hepatology.

[65] Xu L, Hui AY, Albanis E, Arthur MJ, O’Byrne SM, Blaner WS (2005). Human hepatic stellate cell lines, LX-1 and LX-2: new tools for analysis of hepatic fibrosis. Gut.

[66] Bradford MM (1976). A rapid and sensitive method for the quantitation of microgram quantities of protein utilizing the principle of protein-dye binding. Anal Biochem.

[67] Hussain RF, Nouri AM, Oliver RT (1993). A new approach for measurement of cytotoxicity using colorimetric assay. J Immunol Methods.

[68] Pfaffl MW (2001). A new mathematical model for relative quantification in real-time RT-PCR. Nucleic Acids Res.

