# Supplementary figures and images for: Cytoglobin expression in the hepatic stellate cell line HSC-T6 is regulated by extracellular matrix proteins dependent on FAK-signalling
Source: Fibrogenesis Tissue Repair. 2015 Aug 21;8:15. doi: 10.1186/s13069-015-0032-y (PMC4546255; doi:10.1186/s13069-015-0032-y)

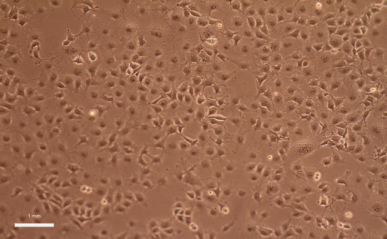


**A**


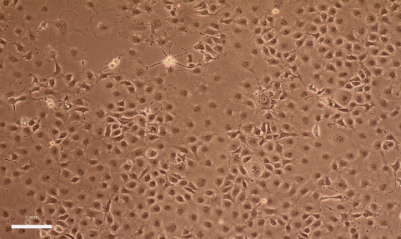


**B**


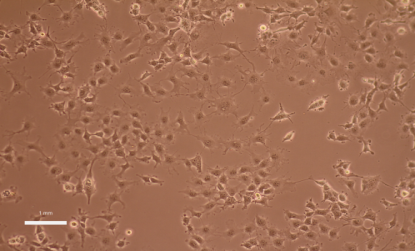


**C**

**Figure S1**

Supplement: Additional file 1: Figure S1. — LX-2 cell morphology on different ECM proteins. Cells were seeded at 300,000 cells/ml imaged 48 hrs post seeding at 100x magnification. A) Non-Coated. B) Collagen I. C) Laminin. (DOCX 729 kb) [file 13069_2015_32_MOESM1_ESM.docx]

**
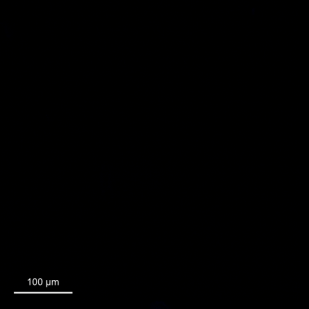
** **

**
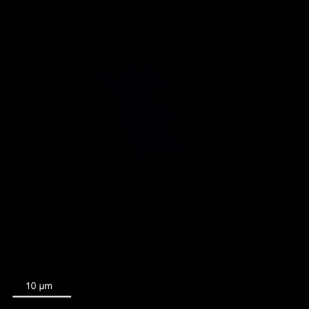

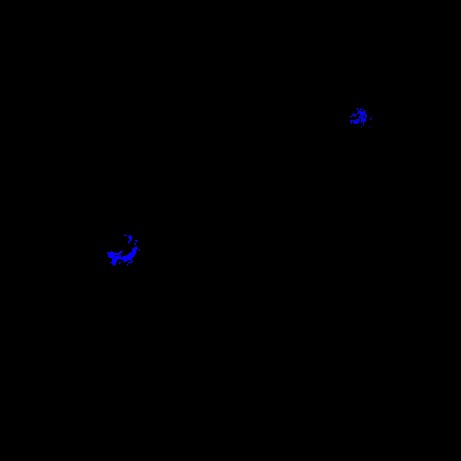

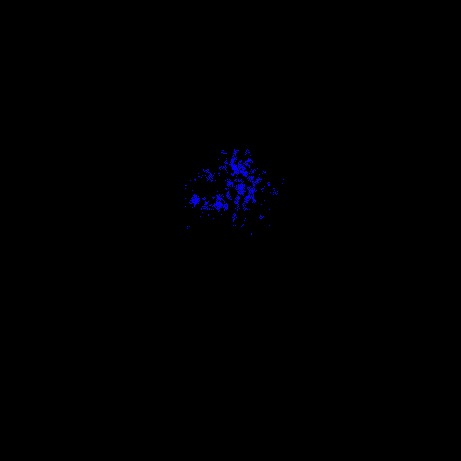


**E**

**D**

**C**

**B**

**A**

**Figure S3**

Supplement: Additional file 3: Figure S3. — Confocal Images of LX-2 cells treated with ATRA cultured on glass, collagen I and laminin 48 hrs post seeding at 300,000 cells/ml, blue—retinoid autofluorescence activated at 351 nm and detected at 515 nm. A) glass, B) collagen I, C) collagen I zoom, D) laminin, E) laminin zoom. (DOCX 260 kb) [file 13069_2015_32_MOESM3_ESM.docx]

**A**

Collagen I coated

Non - coated

**FAK I concentration**

**
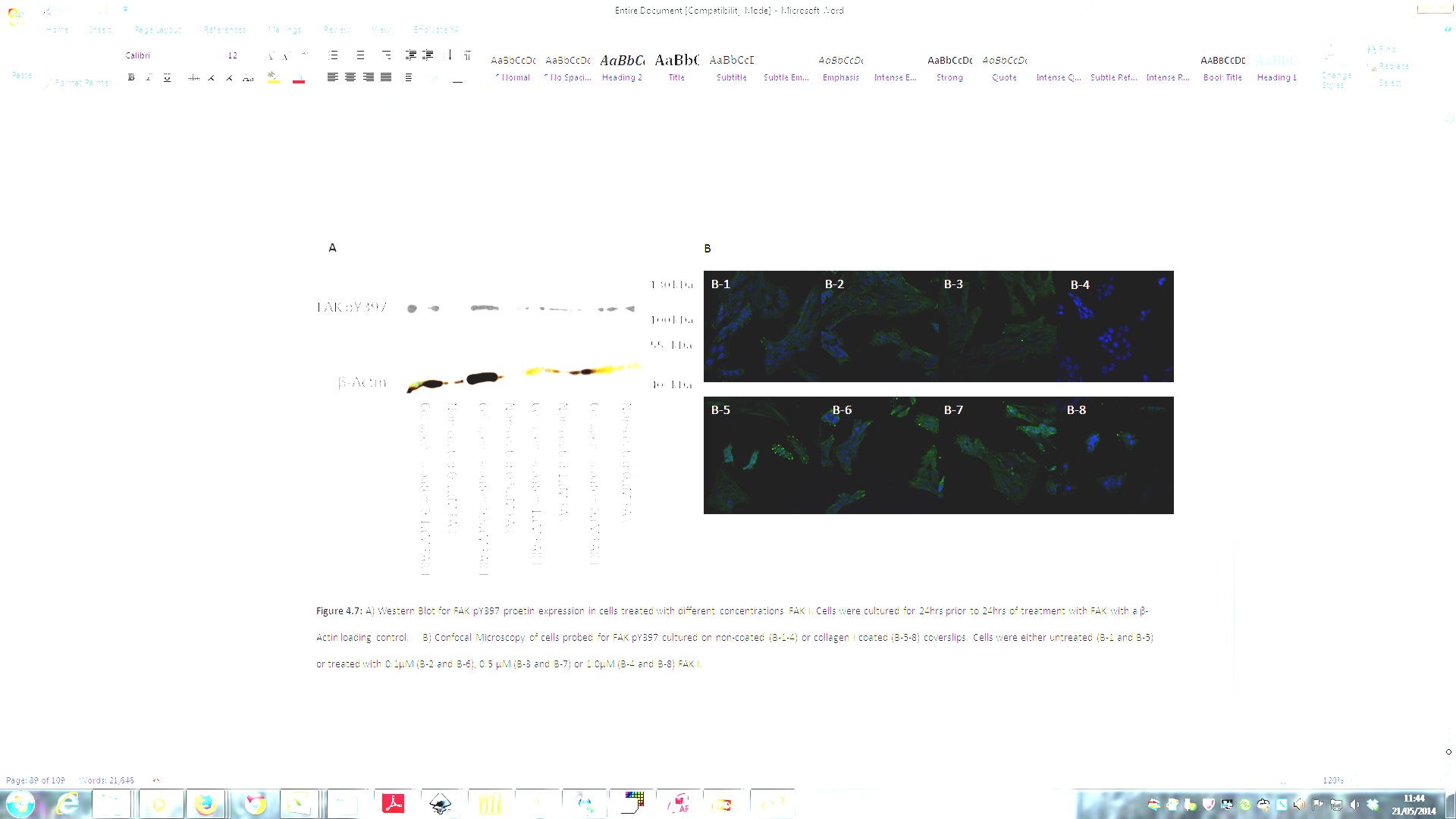
**

0.5 µM

1.0 µM

0.1 µM

0 µM

-4

**Figure S6**

Supplement: Additional file 7: Figure S6. — A) Median flouresence of FAK pY397 analysed by flow cytomtery in cells treated with different concentrations FAKI 14. Cells were seeded at 100,000 cells/ml cultured for 24 hrs prior to 24 hrs of treatment with FAK I then probed for FAK pY397. The results represent the mean of three experiments ± SD. B) Confocal Microscopy of cells probed for FAK pY397 cultured on non-coated (B-1-4) or collagen I coated (B-5-8) coverslips. Cells were either untreated (B-1 and B-5) or treated with 0.1 μM (B-2 and B-6), 0.5 μM (B-3 and B-7) or 1.0 μM (B-4 and B-8) FAKI 14. (DOCX 88.9 kb) [file 13069_2015_32_MOESM7_ESM.docx]
