# Supplementary material for: Cytoglobin expression in the hepatic stellate cell line HSC-T6 is regulated by extracellular matrix proteins dependent on FAK-signalling
Source: Fibrogenesis Tissue Repair. 2015 Aug 21;8:15. doi: 10.1186/s13069-015-0032-y (PMC4546255; doi:10.1186/s13069-015-0032-y)
Supplement: Additional file 2: Figure S2. — Proliferation of LX-2 cells on different ECM proteins. A) Cells were counted using a haemocytometer and counts were taken at 48 hrs post seeding. Cell density is expressed as cells per T25 flask. The results represent the mean of three experiments ± SD. B) Cells from the same population were seeded at 300,000 cells/ml, 1 ml per well and grown on non-coated plastic, laminin or collagen I. Cell number was estimated by the Cell-IQ analysis software. The results represent the mean of three experiments ± SD. (DOCX 53.1 kb) [file 13069_2015_32_MOESM2_ESM.docx]

**B**

**A**

**Figure S2**
