# Supplementary material for: Cytoglobin expression in the hepatic stellate cell line HSC-T6 is regulated by extracellular matrix proteins dependent on FAK-signalling
Source: Fibrogenesis Tissue Repair. 2015 Aug 21;8:15. doi: 10.1186/s13069-015-0032-y (PMC4546255; doi:10.1186/s13069-015-0032-y)
Supplement: Additional file 5: Table S1. — Details Of hydrolysis probe based qPCR assays used in this study. (DOCX 40.6 kb) [file 13069_2015_32_MOESM5_ESM.docx]

| **Gene** | **Species** | **Company** | **Forward (Sense)** | **Reverse (Anti-sense)** |
| --- | --- | --- | --- | --- |
| Cytoglobin (Cygb) | Rat | Applied Biosystems | n/a Unique Assay ID: Rn00590627_m1 | |
| α Smooth Muscle Actin (αSMA) | Rat | Applied Biosystems | n/a Unique Assay ID: Rn01759928_g1 | |
| Hexokinase 1 (HK1) | Rat | Applied Biosystems | n/a Unique Assay ID: Rn00562436_m1 | |
| Focal Adhesion Kinase (Ptk2) | Rat | Primer Design Ltd | AGTGAAGACAAAGACAGGAAAGG | GATCAGGTCCAGCCATGTTCTC |
| Discoidin Domain Receptor 1 (DDR1) | Rat | Primer Design Ltd | TGATTACAGACTACATGGAGAACG | GGAAGCCCCTGAGTGACC |
| Discoidin Domain Receptor 2 (DDR2) | Rat | Primer Design Ltd | TCATCCTGCTGGCTGTCAT | GCTAACTGTCATTTCATCATCCA |
| Integrin beta 1 (β1) | Rat | Primer Design Ltd | TCTGATGAATGAAATGAAATGAGGAGGAT | TTGCTGGTGTTGTACTAATGTATG |
| Integrin Beta 3(β3) | Rat | Applied  Biosystems | n/a Unique Assay ID: 00596601_m1 | |
| Integrin beta 4 (β4) | Rat | Primer Design Ltd | CAGGGTGGAGAAGACTACGA | ACCAGGTGCTCAGTGTCAT |
| Integrin alpha 2 (α2) | Rat | Primer Design Ltd | CAGCACCAGTTTCTTGAAGGA | GCGAACCAACAATCACATCATT |
| Integrin alpha 5 (α5) | Rat | Applied  Biosystems | n/a Unique Assay ID: 01761831_m1 | |
| Integrin alpha 6 (α6) | Rat | Primer Design Ltd | CCGCCGCTCAGAATATCAAG | CCACCACGCTATCCCTGAA |
| Integrin alpha 11 (α11) | Rat | Primer Design Ltd | CCTCTGCTTCGGACCTATCTT | CCGTGGCATATACCGTCTCT |

**Table S1**
